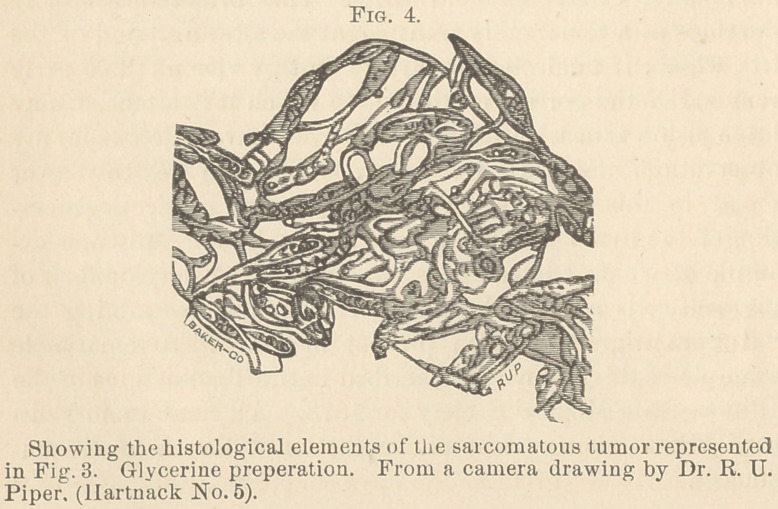# Intra-ocular Tumors

**Published:** 1877-08

**Authors:** E. L. Holmes


### THE PATHOLOGICAL TRANSACTIONS OF THE CHICAGO MEDICAL SOCIETY.

Edited by DR. I. N. DANFORTH.

### I.

### INTRA-OCULAR TUMORS.

(WITH NINE SPECIMENS.)

BY PROF. E. L. HOLMES.

The moment the presence of a tumor within the globe is recognized, there is almost absolute certainty that vision in that eye will be more or less speedily, but in the end totally destroyed; and in the majority of cases, death will be the ultimate result. These tumors, therefore, are nearly all malignant,—such is the nature of the nine specimens which I present for your inspection this evening.

To dismiss with as few words as possible the subject of non-malignant intra-ocular growths, I will simply state that they are chiefly: First: Cysts of the iris. This formation generally develops as a remarkably transparent sac after a wound of the iris, which, if neglected, is sure to destroy vision. The early removal of the portion of the iris to which it is attached, may leave vision intact. But three such cysts have fallen under my observation. Second: Cysticerci, which are scarcely ever found in this country. Third. A peculiar cystic degeneration of the hyaloid artery in the vitreous humor, only one example of which I have ever seen. Fourth: A development of pigment cells around the edge of the pupil resembling the "corpora nigra" so often present in horses. A remarkable example of this anomaly I described in the Transactions of the Illinois State Medical Society for 1873. All these tumors disturb vision more or less under the most favorable circumstances.

The malignant tumors are almost wholly confined to two classes—sarcoma of the choroid, ciliary processes and iris, peculiar to adults, and glioma of the retina, only found, with rare exceptions, in infants or very young children. A very few other forms of tumor with unusual cell structure have been reported in ophthalmic literature.

The sarcomatous tumors before you, with one exception, scarcely fill half of the globe.

Fig. 3.

Choroidal Tumor (Sarcoma). To Illustrate Dr. Holmes' Article.

The eyeball is shrivelled and rendered elliptical by being mounted upon a glass slip suspended in alcohol; but the position and relations of the tumor are well shown.

They vary much in color, from a light brown to jet black. They are characterized by a mixture of small round or elongated and large fusiform or stellate cells, "distinguished in general by large and sharply defined nuclei and bright well marked nucleoli.

Fig. 4.

Showing the histological elements of the sarcomatous tumor represented in Fig. 3. Glycerine preperation. From a camera drawing by Dr. R. U. Piper, (Hartnack No. 5).

The course of development of these growths may be conveniently divided into three periods: 1st. When there is no pain, nor inflammation, when sight in a portion of the field of vision may still be quite good and when the tumor, in uncomplicated cases, may be seen with the aid of the ophthalmoscope. 2d. When inflammatory action has caused closure of the pupil and cataract, and pain, which sometimes causes the patient to seek medical aid. Increased tension (hardness) of the globe in this stage is usually a most important symptom. The diagnosis is sometimes difficult in aged patients, who cannot give the past history of their case, when the symptoms may be those of a neglected glaucoma. 3d. When the tumor has invaded the neighboring tissues.

With the general history of the case, I think the presence of large tortuous vessels in a circumscribed portion of the ocular conjunctiva, is an important symptom, in doubtful cases.

The malignancy of these tumors is scarcely apparent to the general practitioner, when the specialist has extirpated the globe in their early stage of development, for they seldom reappear in the orbit. Death is none the less certain, I believe, although it may take place at a period seldom more than four years subsequent to the extirpation of the globe, from malignant disease of some internal organ, especially the liver. The family physician too often fails to associate the death of his patient with the eye removed some years before.

When the tumor has been permitted to break through the sclerotic and cornea and invade the adjoining tissues, the malignancy becomes apparent. Death may occur from direct injury to the brain, or from excessive discharges, or the absorption of poisonous fluids into the blood.

If we turn to gliomatous tumors we find a growth which, with or without treatment, almost universally and quite speedily forces upon the mind of the general practitioner the idea of malignancy—for whether it is removed or permitted to remain, it rapidly becomes a fungus hæmatodes, destroying life by direct injury of the brain or by exhaustion produced by great discharge or by contamination of the blood.

In nearly all instances the disease appears in the eyes of infants or very young children, who cannot call attention to their failing sight; consequently, the ophthalmologist seldom has an opportunity of examining the tumor in its very earliest stage. When the retina has become involved to a considerable extent, there is clearly seen deep in the eye a peculiar glimmer, which may be compared to polished German silver partially tarnished. This change is usually first observed by the mother of the patient. The diagnosis in the first stage is remarkably simple, although in very rare cases a peculiar form of choroiditis may be mistaken for retinal tumor. It is especially worthy of attention that detached retina, or choroiditis, or in fact any form of disease which might possibly be regarded as glioma, almost never appears in infants. Occasionally glioma is observed in both eyes.

The cells of this growth are characterized by their great similarity to the true granular cells of the normal retina, being round, nucleated, with nucleoli. The term glioma was originally applied to these tumors as developing from the connective tissue (glia, glue), of the retina. As they now seem to be a proliferation of the true retinal granules, the term is a misnomer.

The only treatment of this form of tumor is as early as possible to extirpate the globe. The only object of this operation is to prevent or relieve pain, for it can scarcely be said to save life.

The subject of intraocular tumors has been extensively discussed in especial works and journals of ophthalmology. The best and most available special work for the American reader is perhaps that of Dr. Knapp, of New York.

Reported March 12, 1877.

## Figures and Tables

**Fig. 3. f1:**
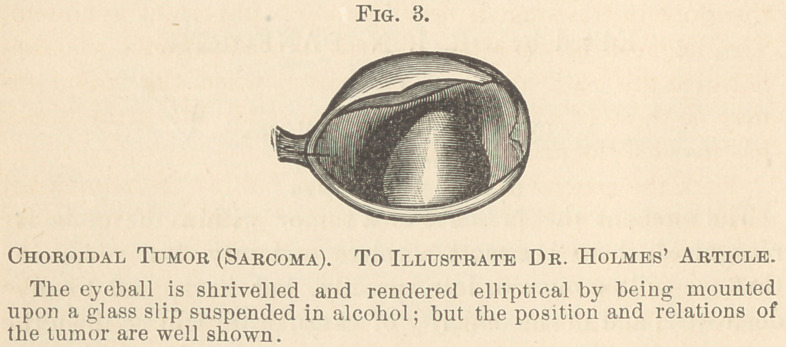


**Fig. 4. f2:**